# IUC24360-80 Second-line therapies of metastatic renal cell carcinoma (mRCC) after first-line immune-combinations (ICI-combos) (Meet-URO 33 study)

**DOI:** 10.1093/oncolo/oyaf248.042

**Published:** 2025-08-29

**Authors:** D Bimbatti, C Messina, L Bonomi, S Scagliarini, S Chiellino, B A Maiorano, F M Deppieri, A Cavo, V Conteduca, S E Rebuzzi

**Affiliations:** Oncology 1 unit, IOV—Istituto Oncologico Veneto IRCCS, Padova, Italy; Oncology Department, Ospedale Civico A.R.N.A.S, Palermo, Italy; Department of Oncology and Hematology, ASST Papa Giovanni XXIII, Bergamo, Italy; UOC di Oncologia, Azienda Ospedaliera di Rilievo Nazionale Cardarelli, Napoli, Italy; Medical Oncology Unit, IRCCS Policlinico San Matteo, Pavia, Italy; IRCCS Ospedale San Raffaele, Milano, Italy; Ospedale dell’Angelo, AULSS 3 Serenissima, Mestre, Italy; Oncology Unit, Villa Scassi Hospital, Genova, Italy; Unit of Medical Oncology and Biomolecular Therapy, Department of Medical and Surgical Sciences, University of Foggia, Policlinico Riuniti, Foggia, Italy; Medical Oncology Unit 2, Ospedale Molinette Azienda Ospedaliero-Universitaria Città della Salute e della Scienza di Torino, Torino, Italy

## Abstract

**Background:**

The treatment landscape of mRCC has deeply changed with the advent of first-line immuno-combinations but most patients inevitably progress. Unfortunately, no standard second line is established, and the heterogeneity of the first line increases this treatment uncertainty. In this context, large-scale real-world evidence is increasingly needed.

**Methods:**

The Meet-URO 33 is a multicentric prospective observational study enrolling mRCC patients receiving first-line systemic therapy. A retrospective cohort of patients treated from January 2021 was included. Analysis on the second systemic treatment line was conducted.

**Results: A total of:**

1,557 patients were enrolled from 52 centers, 407 (26%) started a second^-^line therapy, including 325 (80%) who received first-line ICI-combos. According to the ICI-combo type, 36% of patients treated with Pembrolizumab + Axitinib, 32% with Nivolumab + Ipilimumab, 11% with Nivolumab + Cabozantinib and 9% with Pembrolizumab + Lenvatinib started a second-line therapy. Among progressed ICI-combos patients, 293 (90%) started TKI (mostly Cabozantinib), 25 (8%) drug combinations (mainly Lenvatinib + Everolimus/Belzutifan) and 7 (2%) experimental ICI. Particularly, among progressed IO-IO patients, 88% received TKI and 5% drug combinations, while among progressed IO-TKI patients 91% received TKI and 8% drug combinations. The multivariable analysis showed that younger and poorer Meet-URO score patients (< 0.05) received more second-line drug combinations than TKI. In addition, second-line Cabozantinib after IO-TKI was associated with a modest and non-significant OS difference compared to after IO-IO (HR 0.78, 95% CI, 0.53-1.15; *P* = .20).

**Conclusions:**

The Meet-URO 33 study showed a large-scale real-world evidence of second-line therapies after first-line ICI-combos. The most frequent therapy is Cabozantinib, which showed OS effectiveness regardless of the type of first-line ICI-combos, similarly to other evidence (eg, CaboPoint and CARINA studies).

Drug combinations (Lenvatinib + Everolimus/Belzutifan) are rising as emerging therapies, especially in younger and prognostically unfavorable patients. Further survival and response analyses are planned.

